# Bovine Serum Albumin Bends Over Backward to Interact with Aged Plastics: A Model for Understanding Protein Attachment to Plastic Debris

**DOI:** 10.1021/acs.est.3c10028

**Published:** 2024-05-29

**Authors:** Margaret M. Elmer-Dixon, Liam P. Fawcett, Emma N. Sorensen, Melissa A. Maurer-Jones

**Affiliations:** † Department of Physics & Astronomy, University of Minnesota, Duluth, Duluth, Minnesota 55812, United States; ‡ Department of Mechanical and Industrial Engineering, University of Minnesota, Duluth, Duluth, Minnesota 55812, United States; § Department of Chemistry and Biochemistry, University of Minnesota, Duluth, Duluth, Minnesota 55812, United States

**Keywords:** plastic pollution, protein attachment, photodegradation, polyethylene

## Abstract

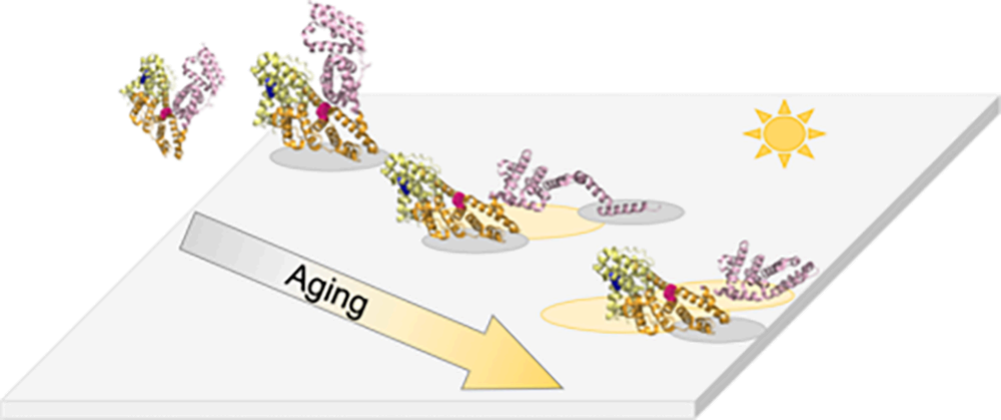

Plastic pollution, a major environmental crisis, has a variety of consequences for various organisms within aquatic systems. Beyond the direct toxicity, plastic pollution has the potential to absorb biological toxins and invasive microbial species. To better understand the capability of environmental plastic debris to adsorb these species, we investigated the binding of the model protein bovine serum albumin (BSA) to polyethylene (PE) films at various stages of photodegradation. Circular dichroism and fluorescence studies revealed that BSA undergoes structural rearrangement to accommodate changes to the polymer’s surface characteristics (i.e., crystallinity and oxidation state) that occur as the result of photodegradation. To understand how protein structure may inform docking of whole organisms, we studied biofilm formation of bacteria*Shewanella oneidensis*on the photodegraded PE. Interestingly, biofilms preferentially formed on the photodegraded PE that correlated with the state of weathering that induced the most significant structural rearrangement of BSA. Taken together, our work suggests that there are optimal physical and chemical properties of photodegraded polymers that predict which plastic debris will carry biochemical or microbial hitchhikers.

## Abstract 2

Polymer weathering changes the structure of proteins as they attach, corresponding changes in biofilm formation, which has implications for the behavior of plastic waste in the environment.

## Introduction

It is well established that anthropogenic plastic waste is problematic for our environment, including ingestion and entanglement caused by bulk plastic debris by charismatic macrofauna.^1^ Plastic also breaks down into smaller, microscopic pieces^2,3^ that have a variety of deleterious effects including impacts to human metabolism^4^ and changes to bacterial secretion^5,6^ upon exposure to nanoplastics and additives. Plastic waste can be deposited directly into the environment as litter via stormwater runoff or carried into the environment from landfills and wastewater treatment plants (WWTPs) by effluent.^3,7^ Indirectly, plastics enter the environment via the degradation of everyday plastic materials (i.e., rubber tires, maritime equipment, polymer coatings, etc.), which then fracture and are carried throughout the environment.^8,9^

While plastic waste is predicted to remain in the environment for centuries,^10^ it is also clear that the plastic waste itself is chemically and physically transformed from a number of environmental stressors, including a primary abiotic pathway of sunlight degradation.^5,8,11−14^ The specific photochemical pathways may vary depending on the polymer molecule, and these reactions can be categorized broadly into oxidation, scission, and cross-linking products.^15,16^ For polyolefins such as polyethylene, which is one of the most commonly observed materials in aquatic plastic pollution, photodegradation yields oxygen-rich functional groups generated within the backbone that decreases its molecular weight while increasing crystallinity.^5,12,17,18^

Polymer chemistry, and the changes in chemistry due to weathering, has shown to impact various behaviors of plastics in natural systems, including the adsorption of organic pollutant compounds.^19^ Beyond plastics acting as a sink for small molecule pollutants,^19,20^ plastics are capable of adsorbing biochemical molecules such as proteins and fatty acids^21^ along with microorganisms^22,23^ that includes biofilm formation. There is evidence that biofilms form on these artificial substrates within hours^24^ and ultimately can influence the fate and transport of microplastics through aquatic systems.^25^ For example, Kaiser et al., shown that biofilms attached to polyethylene and polystyrene increase the sedimentation rate of the microplastics.^26^ Meanwhile, other studies have shown that biofilm formation may aid in the plastic’s ability to transport biotoxins.^25,27^ The studies on the taxonomic structure of these communities vary widely, depending on the natural environment, but some marine studies show microbial species inclined to biodegradation are in higher abundance than in the surrounding waters,^28^ yet another study showed no different community structure within the biofilms in comparing plastic biofilms to biofilms on other solid substrates (e.g., glass or aluminum).^29^ The disparities in these studies highlight the need for fundamental characterization of plastic chemistry and the adsorbed molecules as relates to the propensity for biofilm formation.

The initial step of biofilm formation is adsorption onto the surface. This is a highly complex process that requires both biospecific/selective and nonspecific (hydrophobic or electrostatic) processes.^30^ Typically, the nonspecific repulsion forces must first be overcome. When forming biofilm layers on hydrophilic surfaces, the bacteria will use specific intermembrane, amphipathic anchor proteins called adhesins.^31^ These interacting and adhering proteins form a biomacromolecular layer on the surface that helps to establish a connection between the cell and polymer and overcome any repulsive forces such as incompatibility of the surface and bacteria polarity.^32^ Once the repulsive forces of the surface have been overcome, electrostatic interactions are established, reinforcing the binding between the polymer surface and cell. While biofilm formation can be initiated on both hydrophobic and hydrophilic surfaces, a study by Katsikogianni et al. validated a thermodynamic model that demonstrated that the surface characteristics of both the substrate and bacteria are contributing factors to adhesion.^33^ These findings suggest that the changes to the plastic’s surface due to weathering may attenuate the extent and strength of polymer-cellular interactions. Although biofilms may form on most surfaces, studies have shown that the formation of a protein layer on the substrate surface is generally a prerequisite to the adherence of cells at that site.^34^ The formation of a protein layer is a complex process, but relating protein adsorption to polymer chemistry may be an avenue to understanding larger organism responses that dictate biofilm formation.

As proteins adsorb onto a surface, they can undergo conformational changes that can both affect their structure and potentially influence their function.^35−37^ The work described herein explores the formation of biofilms by sediment bacteria *Shewanella oneidensis* (*S. oneidensis*) on photodegraded polyethylene (PE) and relates the biofilm formation potential to the changes in structure of model protein bovine serum albumin (BSA) as it adsorbs to the PE films. BSA is readily available with a well-documented secondary structure. It is composed of three domains with high alpha helical content (∼67%).^38^ BSA’s highly fluid helical secondary structure is known to respond to changes with pH,^39^ hydrophobicity,^39−41^ and electrostatic composition.^39,41^ Further, the adhesion proteins for the model bacteria also have a helical structure.^42^ Therefore, it makes it a strong candidate to report protein structural responses to environmentally relevant polymer samples and to relate it to bacterial attachment. Ultimately, this work informs the potential response of a flexible protein to an increasingly weathered and the topologically varied polymer surface. Further, we demonstrate the role polymer weathering and surface rearrangement play in creating a preferable interface for growth and proliferation of biofilms in the environment.

## Materials and Methods

### Polymer Preparation and Characterization

Low-density polyethylene 30 μm films were purchased from Goodfellow Cambridge Limited (Huntingdon, UK) and prepared as previously described^5,12^ (see the Supporting Information for more details). Polymer films were irradiated with UVC light (∼254 nm) for either 12, 24, and 48 h on both sides to ensure uniform degradation, which has previously been measured to be ∼6x higher irradiance than noon sunlight near summer solstice at 46.7°N^11^ and causes transformations 3 orders of magnitude faster.^12^ Film age time was reported to reflect the exposure time per side.

Irradiated films were characterized with infrared spectroscopy (FTIR) and differential scanning calorimetry (DSC). FTIR spectra were collected with a Nicolet iS50 FTIR spectrometer (Thermo Scientific, Waltham, MA) with a diamond crystal ATR, and spectra were analyzed with IGOR Pro 8 software (Wavemetrics, Inc., Portland, OR) to calculate the carbonyl index^43^ where the carbonyl region (∼1725 cm^–1^) was normalized to a stable C–H backbone stretch (∼1380 cm^–1^). Additionally, OMNIC (Thermo Scientific, Waltham, MA) was used to calculate surface crystallinity of the PE samples as previously described,^5,11,12^ where the crystalline band (1472 cm^–1^) was normalized by the sum of amorphous stretches (∼1456–1466 cm^–1^). For both carbonyl and surface crystallinity, spectral data were analyzed individually with measurements calculated in triplicate and reported as the average with standard deviations. DSC thermograms were collected with Discovery 250+ DSC (TA Instruments, New Castle, DE). The enthalpy of melting was converted to a percent crystallinity using the value of 293 J/g extrapolated from 100% crystalline samples.^44^ Triplicate samples were analyzed, and crystallinity values are reported as averages with standard deviation. Scanning electron microscopy imaging was performed with a JEOL JSM-6490LV with samples sputter-coated with ∼10 nm Au.

### Protein Preparation

Lyophilized bovine serum albumin (BSA (98%)) was purchased from Sigma-Aldrich (St. Louis, MO). Solutions were prepared in 0.01 M sodium phosphate (PBS) buffer at pH 7.0. BSA solutions were prepared to 7 μM for fluorescence and CD experiments. The protein structure is shown in Figure S1.

### Circular Dichroism (CD) and Absorbance Measurements

CD measurements of BSA were performed on an Applied Photophysics Chirascan V100 (Beverly, MA) equipped with a Quantum Northwest TC1 Temperature Control (Liberty Lake, WA) and a S&A CW-3000 Industrial Chiller (Guangzhou, China). Scans of the protein were performed from 250 to 200 nm (1 nm step size) being held at a constant 20 °C in a Hellma 0.5 mm path length demountable rectangular quartz cell (Plainview, NY) with the light passing through protein solution then plastic sample and finally to the detector. CD spectra were acquired every 5 min for 60 min, and data acquisition was performed in triplicate. Background scans of PBS buffer, in the case of BSA, were taken, and scans of plastic and buffer were taken to be subtracted from the protein measurements. Note that film orientation was set such that the bias, or direction of formation during the blowing process, is perpendicular to the surface of the lab bench to eliminate age-dependent PE-light interactions.^5^ Associated sum absorbances were reported during CD acquisition and allowed us to monitor evidence and propagation of scattering due to protein aggregation.

#### CD Data Processing

CD spectra were individually filtered using a sixth order Saviztky-Golay (SG) filter in Matlab. 230–240 nm slope analysis was performed using a nonlinear least-squares algorithm to extract slopes from individual spectra. Slopes were then averaged for each data point, and error was reported as error in slope fit value. Fractional helicity was found following the protocol reported by Wei and colleagues (see the Supporting Information for more details).^45^ All CD spectra were linearly fit independently from 230 to 240 nm using Matlab (R2020b, Mathworks, CA). The resultant slopes were then averaged and converted to fractional helicity. Absorbance data were analyzed directly with no further processing.

### Fluorescence Spectroscopy

Fluorescence characterization of protein–polymer interactions using tryptophan (W)-fluorescence was performed on a Horiba Scientific FluororMax-4 spectrofluorometer (Kyoto, Japan) equipped with a Quantum Northwest Temperature Control Turret (Liberty Lake, WA) and Koolance EXT-440 Liquid Cooling System (Auburn,WA). Sorption experiments were performed in triplicate with the plastic secured on the far backside of a rectangular 10 mm Starna Cells quartz cuvette (Atascadero, CA) using a glass insert designed to not interfere in the measurement (Figure S2). Fluorescence of the tryptophan residues was monitored with excitation at 285 nm and emission scanning from 300 to 500 nm (1 nm step size). Slit widths of 1.5 nm were used for both excitation and emission scans. Spectra was acquired at a 400 nm/min scan rate. Measurements were taken using the 4-sample turret with consecutive sampling of the four samples occurring every 30 s followed by a 5 min dwell time. Corresponding data represent a sample acquisition every 7 min for 1 h with temperature held at 20 °C.

#### Fluorescent Spectra Data Processing

All data were analyzed in Matlab R2020b (Mathworks, Natick, MA), and postcollection data-smoothing was processed using a 2-Gaussian filter via nonlinear least-squares fitting algorithm where each spectrum was individually analyzed to enable better peak location detection. Fit data were used to ascertain fluorescence peak location shift information. Center of mass data (COM) were calculated using both fit data and raw data with no deviation in COM calculations for any of the data sets reported.^46^ Second derivative analysis was independently performed on postcollect data smoothed with a sixth order Saviztky-Golay (SG) filter with data initially smoothed followed by a sequential numerical gradient. SG and 2-Gaussian filter results were compared and showed no significant deviation between techniques. See the Supporting Information, including Table S1, for more details.

### Bacterial Biofilm Growth

*Shewanella oneidensis* MR-1 (ATCC, 700550, Manassas VA) was grown on LB broth agar plates at room temperature, and single colonies were inoculated into 10 mL of LB broth to establish the suspended bacteria used for subsequent biofilm work. This suspension was grown at 30 °C and shaken at 200 rpm overnight and used within 24 h post inoculation. The optical density (λ = 600 nm) of the suspension was measured and converted to a cell density (1 AU = 10^8^ cell/mL) to ensure that consistent cell density was delivered to the experimental conditions. PE samples (∼2.5 × 2.5 cm) were placed in a milk dilution bottle with 19 mL of nutrient deficient M4 broth (recipe previously described).^6,47^ Approximately 1.25 × 10^8^ cells were added, and the bottle was sealed for 3 days to induce anaerobic conditions optimal for biofilm growth.

After the 3 days of growth, the plastic samples were removed and rinsed with a PBS buffer to remove planktonic cells before being left to air-dry. Once dry, the samples were stained with crystal violet (1% solution; Fisher Scientific) for 20 min before being rinsed with water and air-dried once again.

#### Biofilm Quantification

The stained samples were placed into a vial with 3 mL of pure ethanol for 45 min to leech the stain from the biofilm. UV–visible absorbance measurements were taken using a Genesys 50 UV–visible Spectrophotometer (1600 nm/min, 1 nm step size; Thermo Fisher, Waltham, MA) from each of the leached samples. The absorbance value at 595 nm normalized to the mass of the sample (in g) was used to quantify biofilm formed on each sample as has been previously described.^48^

## Results and Discussion

### Artificially Aged Films Undergo Physical and Chemical Changes Dependent on Exposure Time

**Figure 1:**
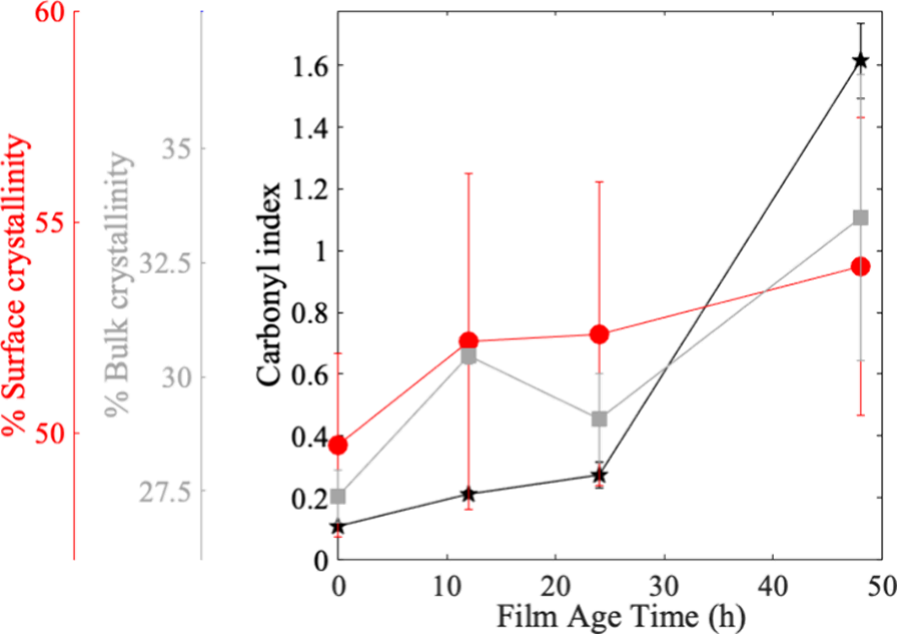
Average percent surface crystallinity (red axis) as measured with FTIR for each film irradiation time (red circles). The average percent bulk crystallinity (gray axis) was measured with DSC for each film irradiation time (gray squares). The average carbonyl index (black axis) as measured with FTIR for each film irradiation time (black stars). Trend lines were added to all data sets for the sake of clarity. Error is represented as standard deviation of mean.

PE samples were characterized for changes in chemical properties to better understand the surface physical and chemical properties that dictated biochemical attachment. Figure 1 and Figure S3 show the films chemically and physically age, where the carbonyl index, a marker of oxidation, and surface crystallinity increase with increased irradiation. Bulk crystallinity also generally increases, although there is a slight decrease from 12 to 24 h, which is a similar trend that we observed previously.^5^ Photodegradation is largely a surface phenomenon at early time points. Therefore, the plateau or decrease in bulk crystallinity while surface crystallinity continues to rise suggests that the surface degradation is dominating the transformation process, shown qualitatively through changes in surface roughness observed in SEM images (Figure S4). Overall, the most degraded surface (48h) has the highest carbonyl index and crystallinity values reflecting significant chemical and physical changes to the polymer.

### Degradation State of Polymers Dictates Biofilm Attachment

**Figure 2:**
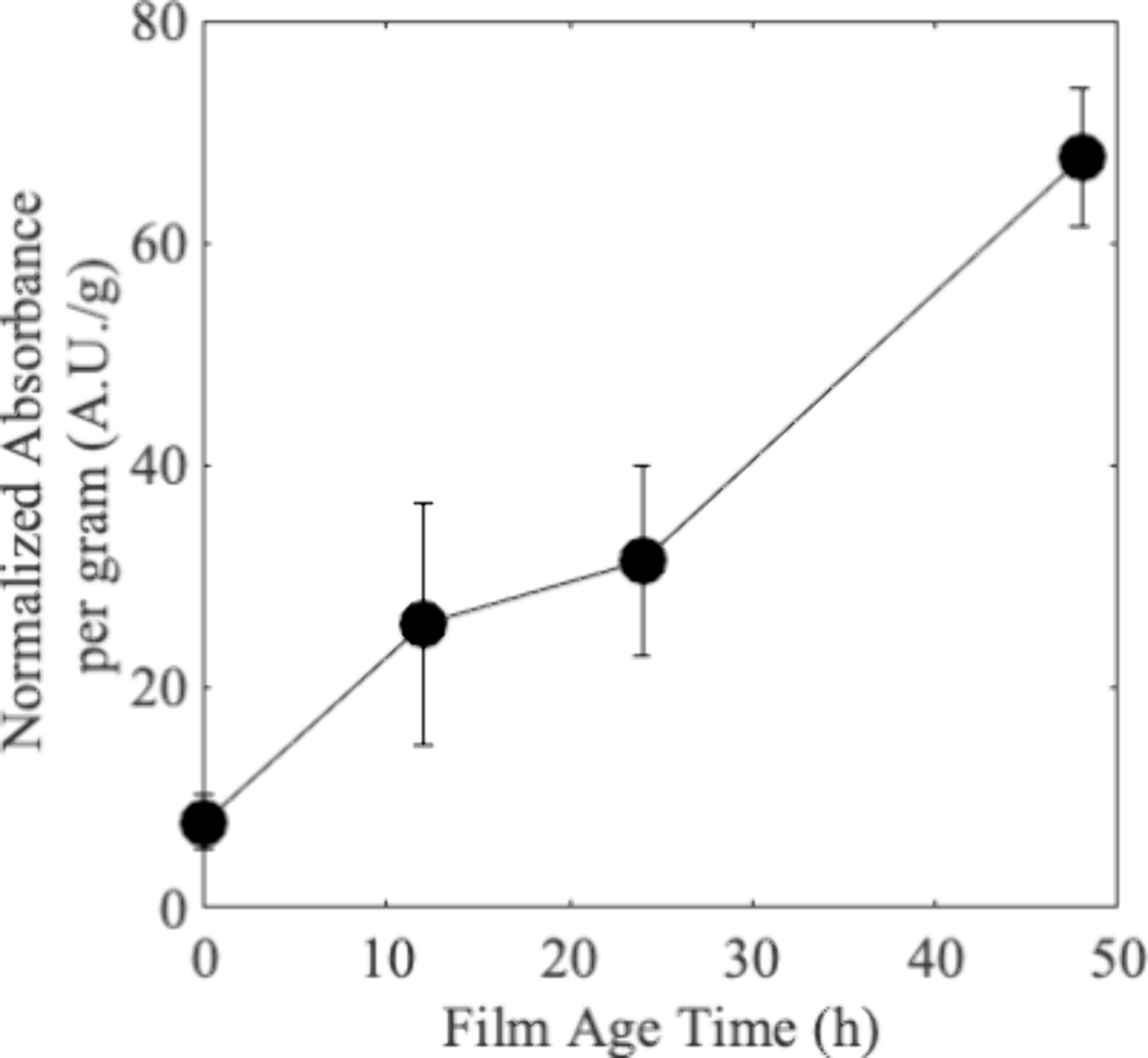
Measurement of the absorbance of biofilm stain crystal violet leached from stained biofilms of *S. oneidensis* and normalized to the mass (in g) of the polymer sample that had irradiated for 0, 12, 24, or 48 h. Markers represent the average of four samples, with the standard deviation reported as the error bars. Lines were added between the markers for clarity.

*S. oneidensis* preferentially forms biofilms on PE with increasing photodegradation as shown in Figure 2 where the biofilm was quantified by measurement of the absorbance of the crystal violet bacterial stain (see Figure S5 for images of stained biofilm). A higher absorbance means a greater biofilm formed and therefore a greater amount of stain taken up on the sample. As it relates to the changes in polymer characteristics shown above, the increased hydrophilicity of the polymer is likely driving adhesion. That is, increased crystallinity has been reported to either decrease the affinity of biological molecules^49^ or not matter for microbial attachment,^50^ so the trend of increased biofilms is concluded to be a response to another material’s property change, such as changes in hydrophilicity. *S. oneidensis* relies on a series of proteins within its genome to effectively produce biofilms. Of these proteins, the adhesin protein BpfA is noted as a necessary part of the biofilm formation process.^42^ Although lacking full characterization, BpfA is analogous to the well characterized adhesin protein from*Pseudomonus fluorescens* LapA,^51^ from which we can make comparisons. From the characterization of the large multidomain protein LapA, it was shown that specific domains are responsible for the proper adherence, where certain domains such as the highly helical von Willebrand factor type A are necessary for the formation of biofilms on hydrophilic surfaces. The trend in biofilm growth seems to match the overall changes in materials characteristics, where there is a more dramatic change in carbonyl and crystallinity from 24 to 48 h irradiation, that may facilitate a prime environment for the strong adherence of multiple domains of the BpfA protein promoting an increased propensity for *S. oneidensis* to form a biofilm on the 48 h degraded films.

### BSA’s Secondary Structure Rearranges upon Interacting with PE

**Figure 3:**
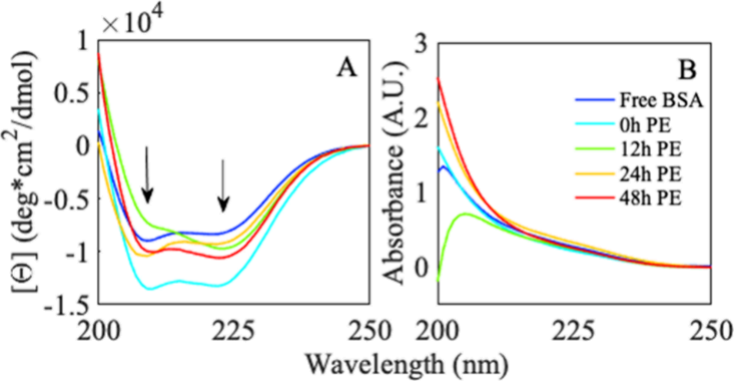
(A) CD spectra of 7 μM BSA in 10 mM phosphate buffer, pH 7 were exposed to (blue) buffered solution, 0 h (cyan), 12 h (green), 24 h (yellow), and 48 h (red) PE. Arrows highlight troughs at 208 and 222 nm. (B) Corresponding sum absorbance spectra of BSA upon initial exposure to (blue) buffered solution, 0 h (cyan), 12 h (green), 24 h (yellow), and 48 h (red) PE. Legend highlights this protein-sample exposure.

To understand how proteins interact with the irradiated polymer surface, we interrogated the changes to the secondary structure of BSA, which models a highly helical attachment domain. BSA’s secondary structure was interrogated with CD spectroscopy. Changes in the classic protein secondary structures are reflected in the resultant difference spectrum and represent global rearrangements in the protein structure (see Figure S1 for the structure). The CD spectrum of free BSA reports the native conformation of the protein when it is in solution (Figure 3A, blue). With this baseline, we then were able to interrogate the effect of polymer films on the secondary structure of BSA. CD spectra showed evidence of an initial protein rearrangement when free protein was exposed to increasingly degraded PE (Figure 3A). Interestingly, this initial response by the protein varies depending on film age with no obvious trend linked to increasing degradation time.

Distinct differences in the CD spectra of exposed proteins reveal insight into the polymer-induced global rearrangement of the protein. A loss of the double trough structure at 208 and 222 nm (Figure 3A, 0 h → 12 h, cyan → green, arrows) generally corresponds to an α helix relaxing and loosening structure. An increase of negative trough at 208 nm (as seen in 12 h → 24 h, green → orange) may report a protein being forced into a tighter, more compact, conformation. Both signal shifts are often seen during protein-binding interactions.^52^

All BSA samples exposed to variably degraded PE report spectra that deviate from the free protein, and these protein spectra are also significantly different from each other. These findings suggest that, while the protein must rearrange to interact with each surface, the conformational change required for the interaction is distinctly related to the degraded surface. The structural rearrangement responsible for shifts in the protein’s initial spectrum occurs within the first minutes of sample preparation and before probing. The experimental error of the replicate cuvettes suggests that these spectral changes describe distinct protein–polymer interaction states. Sum absorbance spectroscopy, where right and left circularly polarized light are added together, can be used to determine if the changes in CD are due to fluctuations in concentration or the protein rearranging.^53^ Corresponding spectra for each protein–polymer system (Figure 3B) show no increase in absorbance at 250 nm (corresponding to aromatic side chain absorbance), indicating that protein concentration is not changing.^54^ Therefore, all changes in CD signals are due to the response of protein interaction with degraded PE.

### BSA Continues to Rearrange in the Presence of PE Over Time

**Figure 4:**
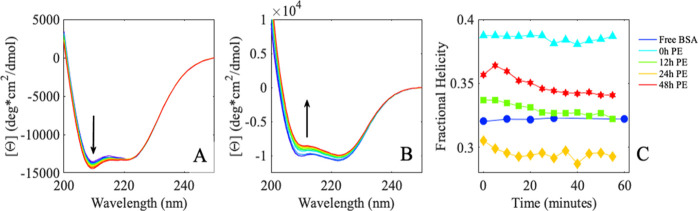
Averaged (*n* = 3) BSA secondary structural CD spectra of BSA interrogated over the course of 60 min upon exposure to (A) nonirradiated PE and (B) photodegraded PE (48 h UV light). All samples were prepared to 7 μM protein in 10 mM phosphate buffer, pH 7. Data were collected every 5 min over the course of 60 min (blue → green → red). Arrows denote CD spectral shifts over the probe time. (C) Averaged slope fit from 230 to 240 nm secondary structural CD data for BSA (A) reported in molar ellipticity (deg × cm^2/dmol/nm) with reported error smaller than marker sizes. Free protein (blue, circle) and protein exposed to 0 h PE (cyan, triangle), 12 h PE (green, square), 24 h PE (orange, diamond), and 48 h PE (red, star) simply connected for clarity. Legend highlights this protein-sample exposure.

BSA was monitored over time to investigate any slower time-scale interaction events between the protein and degraded polymer. CD monitored over the course of 1 h shows a time-dependent change in spectra for all BSA exposed to variably degraded polymer surfaces (Figure 4A,B and Figure S6). The CD signal does not change for free BSA in solution (Figure S6A) over this time course, again indicating that any changes in the spectral signal must be due to interactions with the surface of the polymer.

In each case, the protein’s secondary structure shows a unique response to the polymer based on its specific state of degradation. These changes correspond to either protein compacting, protein loosening/unfolding, or the protein stabilized in final conformation in the presence of the degraded polymer. Specifically, upon exposure to 0 h PE (Figure 4A and Figure S6B), BSA shows an increase in the trough at 208 nm, corresponding to an alpha helical tightening or protein shifting into a more compact structural arrangement. When exposed to 12 h of PE (Figure S6C), BSA experiences an overall loss in helical signal, corresponding to a slight loss in helical content. Twenty-four h PE exposure (Figure S2D) shows no change in helical content, while 48 h PE exposure (Figure 4B and Figure S6E) shows a decrease in the spectral signal at the 208 nm trough indicative of a loss in helical content.

To better understand the polymer-specific protein rearrangement, fractional helicity (Figure 4C) was calculated from these time-resolved spectra of each protein and protein–polymer species. While traditional CD spectral analysis focuses on the presence of troughs, elucidating changes in helical conformation and general unfolding, fractional helicity extracts information from other regions of the spectra and can determine how much of the protein is folded (helical) or unfolded. The associated analysis provides a specific percent helicity for comparison. Together, both traditional CD spectral analysis and fractional helical analysis allow for the determination of the protein conformation and its changes (e.g., unfolding) upon interaction with a polymer.

Fractional helicity analysis shows that, while greater than free protein, there is no substantial changes in helical content for protein exposed to 0 h PE over the 60 min interval of exposure (Figure 4C, cyan). CD data simultaneously demonstrate a global shift in the helical conformation with the pristine plastic (Figure 4A). An increase in a portion of the helical signal reported via the CD signal with no increased percent helicity corresponds to a protein undergoing helical tightening. In fact, a CD signal increase of the 208 nm trough with no evident change in 222 nm trough signal is a spectral signature of helical tightening.^52^

Evidence of protein unfolding is seen in both 12 h CD spectra (Figure S6C) and fractional helicity (Figure 4C, green), strongly suggesting that the protein unfolds to accommodate hydrophilic interactions that occur as the polymer begins to degrade. For the PE photodegraded for 24 h, the protein reports no obvious global structural change (Figure S6D) but shows a decrease in fractional helicity (Figure 4C, orange). This shift may reflect a relaxation of the helical structure at alpha helical end-caps, necessary to accommodate interactions with the degraded polymer surface.^55^ At 48 h PE interaction, BSA shows an increased helicity followed by a decrease in helicity (Figure 4C, red) accompanied by a loss of the alpha helical structure (Figure 4B and Figure S6E). These structural changes may be evidence of a protein binding, rearrangement, and conformational relaxation as the protein adjusts to its final conformation on the polymer surface.

### PE-Degradation-Dependent CD Signal Response Mirrors BSA’s Known Gross Structural Behavior

These motions, taken together, demonstrate the mobility of BSA and aid in understanding the biophysical interactions that are required for a cell to dock onto a polymer substrate. BSA exposed to 0 h PE shows that a cell’s docking proteins may interact and rearrange on an unadulterated polymer surface but will assume a tighter, less optimal conformation with respect to free protein in solution. The extent of degradation of our binding surface enhances the protein’s ability to rearrange and bind to more oxidized surfaces. As the surface continues to degrade, the protein rearranges and starts to unfold into the lowest energy level conformation available. Because proteins function with conformational specificity, it is possible that there is a polymer condition that would enhance the binding affinity of multiple domains and thus initiate a stronger cellular attachment.

### Tryptophan (W)-Fluorescence of BSA is Perturbed by Hydrophobic-PE Surfaces

W-Fluorescence was used to analyze local structural changes upon binding to aged plastics. BSA contains two native tryptophan residues: one buried (W-212) and the other partially solvent exposed (W-134). W-134 sits at the top edge of the protein (Domain I, Figure S1, blue), while W-212 (magenta) sits at the center of the protein in a region (Domain II) well-known to function as a pivot point for structural rearrangement, opening and unfolding.^56−59^ Perturbations to the local environment of tryptophan are known to affect its fluorescence emission spectrum.^60−63^ Shifts from a hydrophobic environment to a more hydrophilic environment correspond to a broadened peak and spectral shift to longer wavelengths. When exposed to water, fluorescence is quenched and, instead of a peak shift, the total fluorescence will decrease. It is important to note that W-fluorescence reports only on the local rearrangement of the protein surrounding the probe.

**Figure 5:**
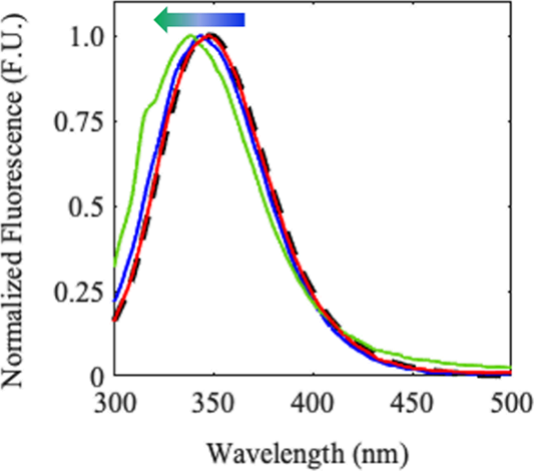
Initial, averaged, normalized tryptophan fluorescence spectra of free BSA (long dashed black line) and BSA exposed to 0 h (blue), 12 h (green), and 48 h (red) degraded PE. The spectrum for BSA with 24 h irradiation PE mirrors the 48h spectrum and was omitted for clarity of the figure.

**Figure 6:**
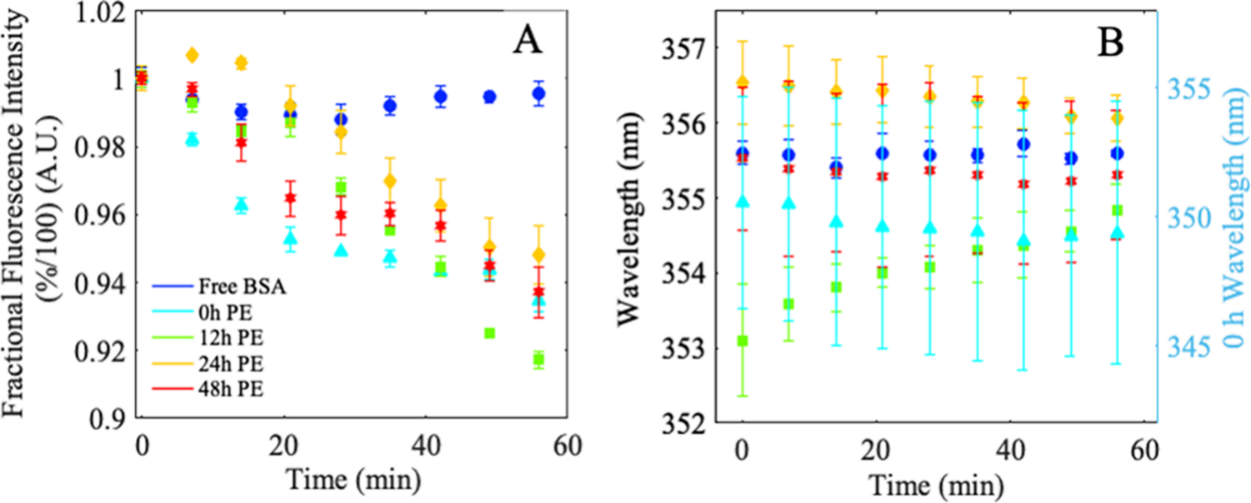
Average percent total fluorescence intensity (A) and average shift in COM (B) tryptophan fluorescence spectra of free BSA (long dashed line blue, circles), and BSA exposed to 0 h (cyan, triangles), 12 h (green, squares), 24 h (yellow, diamonds), and 48 h (red, stars) degraded PE. Legend highlights this protein-sample exposure. Right axis in panel B for BSA exposed to 0 h PE (cyan, triangles).

Like preliminary CD studies, the initial fluorescence signals demonstrate a dependence on polymer aging. Figure 5 shows that initial protein–polymer interactions with 0 (blue) and 12 h (green) PE have a signal shift (blue shifting, direction represented with blue-green arrow in figure) to shorter wavelengths with respect to free protein (long dashed black line). Corresponding COM calculations of the spectra are reported in Figure 6B and discussed later. BSA exposed to more aged plastic (48 h (red) polymer and 24 h polymer (not shown for clarity)), respond with a protein rearrangement generating a more native BSA fluorescence indicative of the tryptophans being reburied in a more hydrophobic environment.

### W-Fluorescence Shows no Local Impact as BSA Rearranges to Interact with PE

All BSA W-fluorescence spectra decrease in fluorescence intensity signal over time when in the presence of PE with no obvious overall shift in the fluorescence peak location over the course of measurement. This indicates rearrangement that leads to hydration induced quenching (Figure S7). Because both tryptophans are relatively buried in the folded conformation, second derivative analysis (SDA) can be used to better understand their individual contribution to bulk fluorescence during protein–polymer interactions (Figure S8). SDA showed that bulk fluorescence for all BSA-PE species is a result of two fluorescent subpopulations, with (1) a major population centered at 350 nm and (2) a minor population at 380 nm. The difference spectra of each SDA used to extract the effect of PE on fluorescence over probe time (Figure S8) revealed no obvious movement by the 350 nm fluorescent populations away from the 350 nm line location during the duration of each experiment. This suggests that the area around W-134 is not susceptible to enough structural rearrangement to permit fluorescence quenching in the presence of water. This local environment is stable for BSA exposed to variably aged plastic suggesting that Domain I (Figure S1, yellow) is not the primary location for protein rearrangement during polymer interaction. Like W-134, W-212’s 380 nm fluorescent signal does not undergo a shift in wavelength location throughout each experiment or between experiments. Interestingly, when compared between aged polymer experiments, the unaged PE and 12 h PE show a decrease in intensity at 380 nm, indicative of W-212 hydration and quenching. Here, the area around the fluorophore does not rearrange to drive a variation in fluorescence but undergoes movement that results in a loss of fluorescence. It can be more clearly stated that, in cases where there is a decrease in fluorescence signal intensity, it is due to quenching upon exposure to water and not a result of stabilized structural rearrangement into a different protein conformation. Fractional fluorescence intensity and center of mass analysis can be used to further extract distinct protein responses to variably changing polymer surfaces.

### W-Fluorescence Reveals Distinct Binding Kinetics Behavior Dependent on the Binding Surface Environment

Fractional fluorescence intensity was calculated for all BSA and BSA-PE species and showed a decrease in percent intensity in all BSA exposed to PE (Figure 6A). All BSA-PE samples show evidence of binding kinetics and can be fit using a Langmuir binding model (see the Supporting Information for more details and analysis).

BSA has been shown to relax into more favorable conformers previously. Zhang and colleagues reported slower protein dynamics that resulted in structural rearrangement from α helix/beta turn toward β sheet during the first 2 h of experimentation.^59^ A shift in the local environment around either W-134 or W-212 would likely expose the fluorophore to a disordered water environment, resulting in a decrease in bulk fluorescence. Time-dependent evaluation of the fluorescence intensity may reflect protein breathing and rearrangement after initial docking. Where changes in the surface chemical and physical structure drive the requisite changes for a protein to adsorb to the surface.

### W-Fluorescence Decreases Over Time for All Populations Exposed to PE

The calculated COM of fluorescence showed a slight signal shift to lower wavelengths over time for BSA exposed to 0, 24, and 48 h PE (Figure 6B, cyan, orange, and red). In concert with the decrease in fluorescence, the signals shift to lower wavelengths that points to W-212 becoming more hydrated and quenched. These observations are in agreement with a BSA protein opening up around the center of the protein (Domain II) and shifting from the “V’ shaped” native conformation to an energetically more favorable open conformation. BSA exposed to 12 h PE experienced an increase in the center of mass corresponding to an increase in W-212 fluorescence with respect to W-134.^63^ This may result from W-134 (at the top of the protein) becoming water exposed and quenched as a result of unfolding around the edge of the protein as opposed to pivoted opening around the core.

### BSA Morphs to Bind to Plastics and Points to Protein Behavior that Promotes Biofilm Formation

The protein motions observed here through CD and W-fluorescence spectroscopies mirror well-known environmentally dependent morphological behavior (see summaries in Tables S2–S4). Upon exposure to 0 h PE, BSA undergoes subtle rearrangement into a favorable conformation to initiate protein interaction. Helical tightening can pack and shield external hydrophilic amino acids and expose core hydrophobic amino acids, introducing these once core amino acids to a more favorable environment, much like that of a hydrophobic polymer surface. It is unsurprising to see signs of helical tightening in the BSA CD signal. *In silico* studies have pinpointed preferred areas of surfactant interaction that would be easily exposed during helical tightening.^64^ Further, *in silico* studies of protein secondary structures and nanoparticles of polyethylene predict this helical stabilization.^65,66^ These regions specifically show interactions around W-212. Fluorescence data confirm protein rearrangement with a decrease in fluorescence over time and a COM blue shift, both indicative of W-134 becoming more buried or W-212 hydrating and quenching as the protein binds to the hydrophobic surface, in agreement with predictive modeling.

PE aging elicits a more dynamic protein response dependent on both the physical and chemical properties of the aged polymer surface. From 0 to 12 h, the interaction surface has incorporated oxygen, introduced bulk and surface crystallinity, and shows signs of preferred cell growth. Fractional helicity calculations report a 7.8% decrease in overall helicity, and peak fluorescence measurements reveal an 8% decrease in fluorescence. Studies of native and open conformers of BSA report a 19% decrease in helical content as the protein opens into a linear conformation, pivoting around Domain II and increasing hydrophobic solvent accessible surface area in all domains.^57^

COM data suggest that a decrease in W-134 fluorescence is responsible for the red shift in mass center to longer wavelengths. These data taken together suggest that helical unraveling occurs in and around W-134, opening Domain I and exposing the fluorophore to water. The SDA of W-212 for this aged film also suggests some hydration of the fluorophore, in alignment with the protein opening into a linear conformation. This new conformation would permit the protein to interact with the primarily hydrophobic surface as well as initiate hydrophilic contacts when possible and given the surface landscape.

BSA exposed to 24 h of PE reports the lowest initial fractional helicity with a further decrease in helicity (∼10%) over the course of the experiment. Interestingly, the same system demonstrated a brief increase in peak fluorescence before experiencing a 6% loss in fluorescence. During the same period, the COM systematically blue-shifts to shorter wavelengths. These data suggest that the protein appears to initially bind to a diverse surface environment and then slowly twists, opening further, into a favorable final conformation that forfeits helical content and native conformation for stability. This supports the notion that the diverse array of adhesin domain structures that adsorb to the surface of the polymer directly influences the strength of biofilm formation, where more dynamic domains preferentially influence adherence through slow structural rearrangements.

Finally, BSA is exposed to an extensively hydrophilic surface, populated with distinctly crystalline regions and providing surface access to incorporated oxygen. This new landscape supports a bound protein with the highest detected helical content for this set of experiments and a protein that shows evidence of independent structural rearrangement around Domains I and II to initiate favorable, stable surface interactions. Simultaneously, this surface is seen to support the most extensive cell growth. This finding suggests that perhaps the hydrophilic and crystalline topology permits the protein to assume a conformation where its motifs or active domains are able to retain the conformational fold required for functionality. For cells that proliferate in primarily hydrophilic environments, it is unsurprising to see an increased level of growth on a surface composed of the favored attributes for cell growth. What is interesting is that, on the macromolecular level, proteins exposed to the same preferred environments show signs of both being unfolded and also retaining the conformational structure, and potentially local functionality, of the unbound protein. This work shows the complicated and fluid nature of protein adherence to polymer surfaces as they begin the degradation process, yielding a better understanding of how our natural environment interacts with the synthetic. Moving beyond adhesion and with the emerging evidence of nanoplastics accumulating in the cells of complex organisms, this work suggests that plastics taken up by animals and humans could impact a protein’s structure and function. Continuing to study how other, more complicated, model proteins interact with these degraded polymers will aid in our understanding of how the taxonomic structures in these environments flux as the plastic waste moves and transforms.

## Supporting Information Available

Additional polymer preparation/characterization and spectroscopic methods and results, fitting parameters for Langmuir fitting and 2- and 3-state fluorescence populations, BSA structure, fluorescence cuvette set up, biofilm images, raw CD and W-fluorescence spectra, and second derivative analysis of fluorescence data (PDF)

## Notes

Plastic pollution can act as a vector for biological molecules and microbial species. Proteins binding to weather polyethylene change the structure in meaningful ways that can inform the binding and formation of biofilms in natural systems.
