# Supplementary material for: Bovine Serum Albumin Bends Over Backward to Interact with Aged Plastics: A Model for Understanding Protein Attachment to Plastic Debris
Source: Environ Sci Technol. 2024 May 29;58(23):10207–15. doi: 10.1021/acs.est.3c10028 (PMC11171446; doi:10.1021/acs.est.3c10028)
Supplement: Supplementary file 1 — es3c10028_si_001.pdf [file es3c10028_si_001.pdf]

# Supporting Information

## Bovine Serum Albumin Bends Over Backward to Interact with Aged Plastics: A Model for Understanding Protein Attachment to Plastic Debris

Margaret M. Elmer-Dixon[1,2]*, Liam P. Fawcett[3], Emma N. Sorensen[3], Melissa A. Maurer-Jones[3]*

**Affiliations:**

[1]Department of Physics & Astronomy, University of Minnesota, Duluth; Duluth, Minnesota, USA, 55812.

[2]Department of Mechanical and Industrial Engineering, University of Minnesota, Duluth; Duluth, Minnesota, USA, 55812.

[3]Department of Chemistry and Biochemistry, University of Minnesota, Duluth; Duluth, Minnesota, USA, 55812.

*Corresponding authors' emails: melmerdi@d.umn.edu and maujones@d.umn.edu

**SUMMARY OF SI CONTENT:**

Pages: 11

Figures: 8

Figure S1. Structure of BSA
Figure S2. Fluorescence cuvette set up
Figure S3 and S4. Additional polymer characterization upon aging
Figure S5. Images of stained biofilm
Figure S6 – S8. Additional spectroscopy characterization of BSA

Tables: 5

Table S1. W-fluorescence fit parameters
Table S2 – S4. Summarizing changes observed in BSA upon polymer property changes with aging

## Additional Methods:

### Polymer Preparation and Characterization

Low density polyethylene 30 μm films were purchased from Goodfellow Cambridge Limited (Huntingdon, UK). Samples were prepared into rectangular pieces and soaked in hexane, methanol, and doubly distilled water for 24 h each to ensure the removal of processing additives and unpolymerized monomers or oligomers. Films were mounted on cardstock and irradiated in a Rayonet merry-go-round photoreactor (Southern New England Ultraviolet Corp, Bamford, CT) using 16 Hg vapor lamps for exposure with 254 nm light.

*Attenuated Total Reflectance-Fourier Infrared Spectroscopy (ATR-FTIR*). Irradiated films were scanned and analyzed using a Nicolet iS510 FTIR spectrometer (Thermo Scientific, Waltham, MA) with a diamond crystal ATR scanning 400-4000 $cm^{-1}$ with a resolution of 4 $cm^{-1}$ and scan averaging of 64 scans per spectra. Backgrounds were taken prior to each new scan and the film was sampled in at least 3 different locations to ensure uniformity of the photochemical transformation over the film's surface area. All spectra were acquired using OMNIC software in triplicate (Thermo Scientific, Waltham, MA)

*Differential Scanning Calorimetry (DSC).* Thermograms of irradiated polymers were collected on a Discovery 250+ DSC (TA Instruments, New Castle, DE). PE samples were cycled from 10 $^{o}C$ to 180 $^{o}C$ at 10$^{o}C$/min in a heat, cool, heat cycle. The melting peak was integrated within the Trios software (TA Instruments, New Castle, DE), and the peak temperature and enthalpy values were recorded.

### Fractional Helicity

The fractional helicity quantifies the structural change in terms of a percentage and provides additional insight into whether a protein is structurally rearranging or unfolding. To better tease out trends in helical structure, fractional helicity was determined using the 230-240 nm slope method of analysis outlined by Wei and colleagues.[1]The resultant slopes were then averaged and converted to fractional helicity (Figure 4 in Main Text). The slope method permits extraction of secondary structural changes in cases where noise prohibits secondary structural elucidation below 230 nm. Essentially, the steeper the curve between 230 – 240 nm, the higher the slope and helical content. A decrease in slope corresponds to a decrease in helical content and represents a loss of secondary structure. The slope method is able to elucidate the loss of alpha helical content but does not distinguish between $3.6_{13}$ and $3_{10}$ helical substructures.

### W-fluorescence Center of Mass (COM) Calculations

Center of mass (COM) data was extracted from individual spectra for each time point in the the fluorescence study and fit following the methodology outlined by Tigner et al.[2] Briefly, the fluorescence signal at each wavelength was weighted by its location and normalized by the sum of all wavelengths reporting fluorescence using the following equation where $x_{nm}$ is the specific wavelength, $m_{nm}$ is the photon count or fluorescence intensity at that wavelength and $X_{com}$ is the resultant location of the center of the fluorescence emission signal

$$X_{com} = \frac{\sum_i (m_{nm} * x_{nm})_i}{\sum_i m_{nm_i}} \quad \text{Eqn S1}$$

**W-fluorescence fitting**

W-fluorescence data were fit with a 1- or 2- layer (or 2- or 3- state) cooperative Langmuir sorption model to extract binding time for protein exposed to variably aged polymer. For the spectroscopic constants $S_o$. $S_1$, and $S_2$, the bulk population of a system can be fit to a 1-layer (2-state) system,

$$\theta(x) = \frac{S_o + S_1 \left(\frac{x}{k_1}\right)^{n_1}}{1 + \left(\frac{x}{k_1}\right)^{n_1}} \quad \text{Eqn S2}$$

Or 3-state system,

$$\theta(x) = \frac{S_o + S_1 \left(\frac{x}{k_1}\right)^{n_1} + S_2 \left(\frac{x}{k_2}\right)^{n_2}}{1 + \left(\frac{x}{k_1}\right)^{n_1} + \left(\frac{x}{k_2}\right)^{n_2}} \quad \text{Eqn S3}$$

Where $k_1$ and $k_2$ are the rates associated with transitions and $n_1$ and $n_2$ is the hill coefficient characterizing the sorption cooperativity and $\theta(x)$ is the detected spectroscopic signal. Fit data reported in Table S1.

*Second derivative Analysis*

While the 330-350 nm emission peak is the most commonly used spectral characteristic of W-fluorescence, there is a secondary emission at 380 nm as well. By taking the second derivative of fluorescence data in this region and comparing over time, it is possible to track if/when a population increases or decreases fluorophores were quenched during interactions with BSA This emission corresponds to how confined the tryptophan is, with a higher signal intensity reporting a more confined fluorophore.

Second derivative analysis clearly demonstrates that fluorescence subpopulations are well established for BSA and may change but do not evolve over the course of experiment making overall intensity max an appropriate technique for further analysis.

**Table S1.** Rate and hill coefficient fit to either 1- or 2- layer (2 state or 3 state) population corresponding to Eqns S2 and S3 for W-fluorescence maximum decrease corresponding to data reported in SI Figure S7A and D.

| | | $k_1$ (minutes) | $k_2$ (minutes) | $n_1$ | $n_2$ |
|---|---|---|---|---|---|
| **BSA** | **0 h** | **10.19** | **42.55** | **1.972** | **6.237** |
| | **12 h** | **34.43** | **--** | **4.11** | **--** |
| | **24 h** | **45.57** | **--** | **2.61** | **--** |
| | **48 h** | **14.36** | **32.96** | **4.812** | **12.52** |

## ADDITIONAL FIGURES:

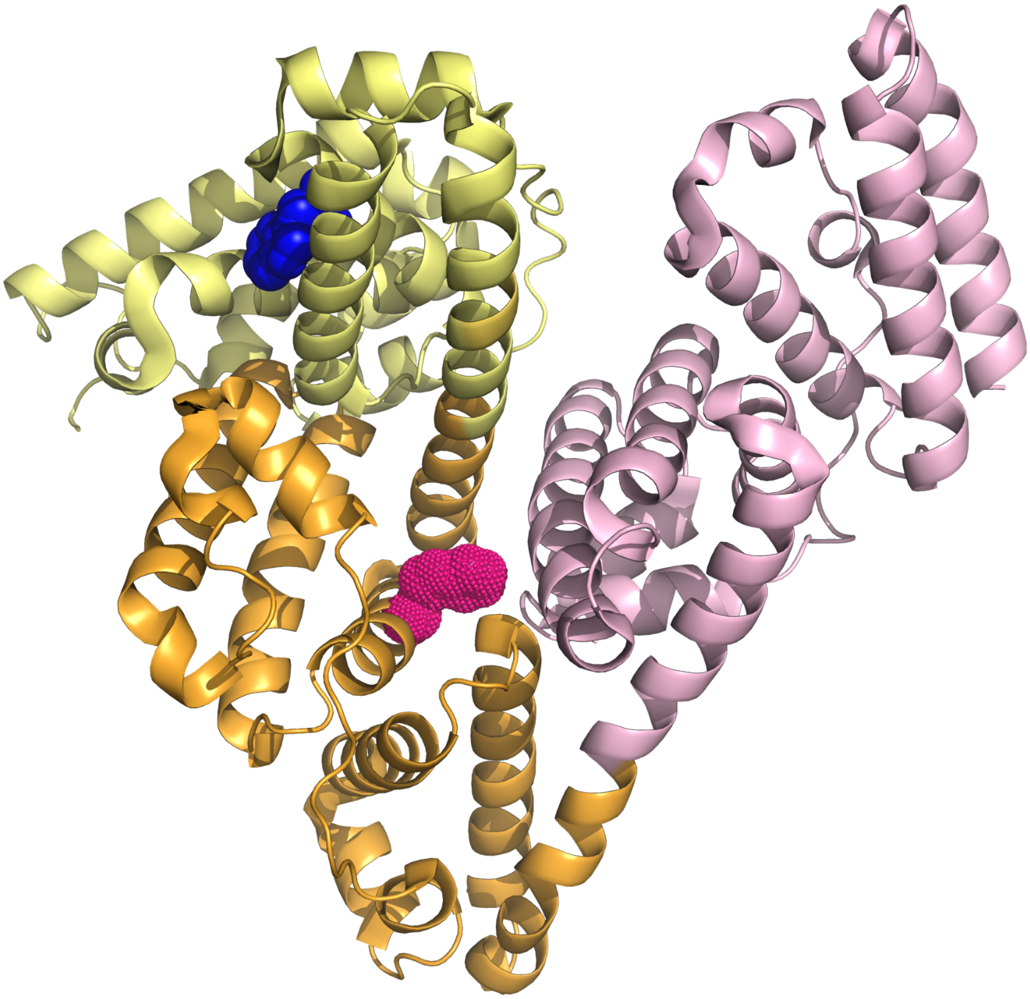

**Figure S1.** PDB 3V03 [3] of BSA model created using MacPyMol, Domains I, II and III (yellow, orange and pink ribbon) highlighted to show locations of Trp-134 (blue) and Trp-212 (magenta) with respect to the interior and exterior of protein.

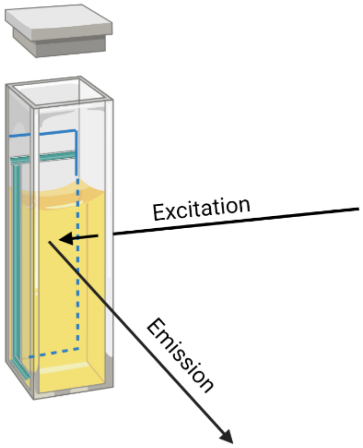


**Figure S2.** Diagram of cuvette set-up for fluorescence binding studies. The light path and the positioning of PE sample (blue) and glass insert (green) are shown. Created with BioRender.com

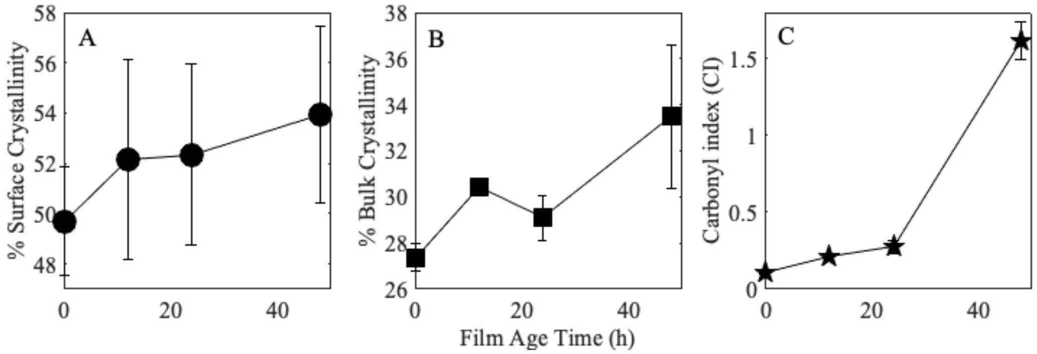


**Figure S3. A:** The average percent surface crystallinity as measured with FTIR for each film irradiation time. **B:** The average percent bulk crystallinity as measured with DSC for each film irradiation time. **C:** The average carbonyl index as measured with FTIR for each film irradiation time. Trend lines were added to all data sets for clarity. Error represented as standard deviation of mean.

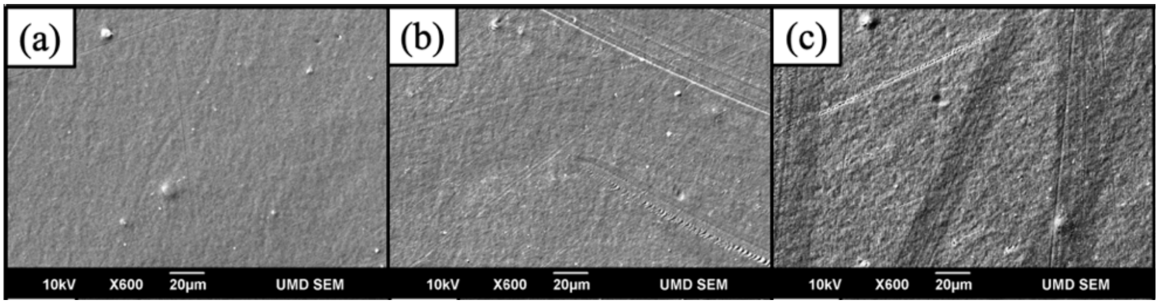


**Figure S4.** SEM images of polyethylene thin films where (a) is pristine, (b) 24 h UV aged, and (c) 48 h UV aged. Qualitative assessment supports the increase of surface roughness with greater weathering.

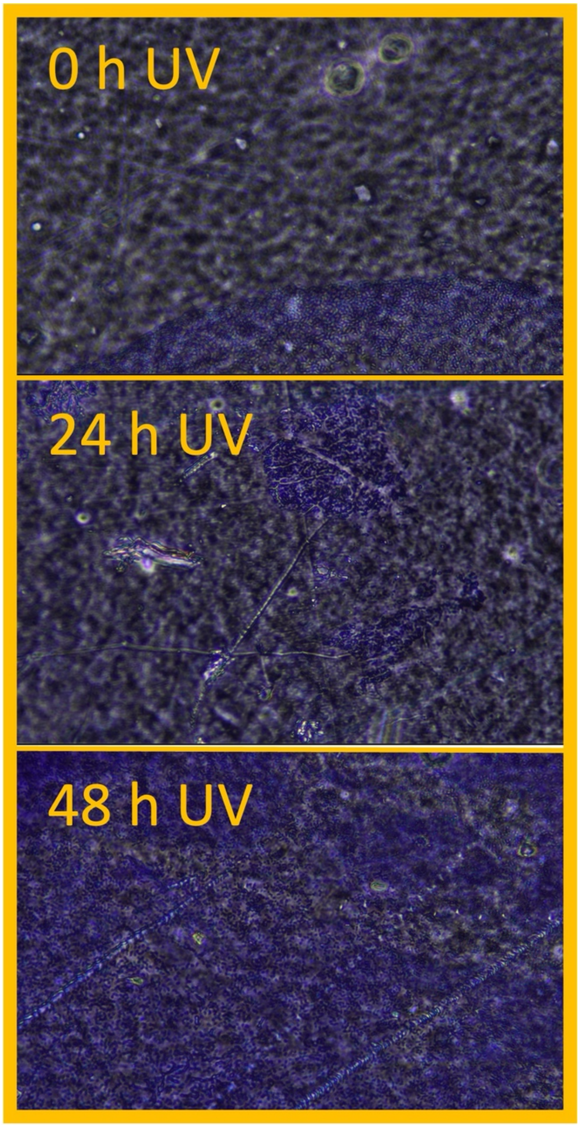


**Figure S5.** Brightfield images of biofilms of *S. oneidensis* stained with crystal violet grown on pristine and UV weathered polyethylene. Biofilms appear as purple within the images.

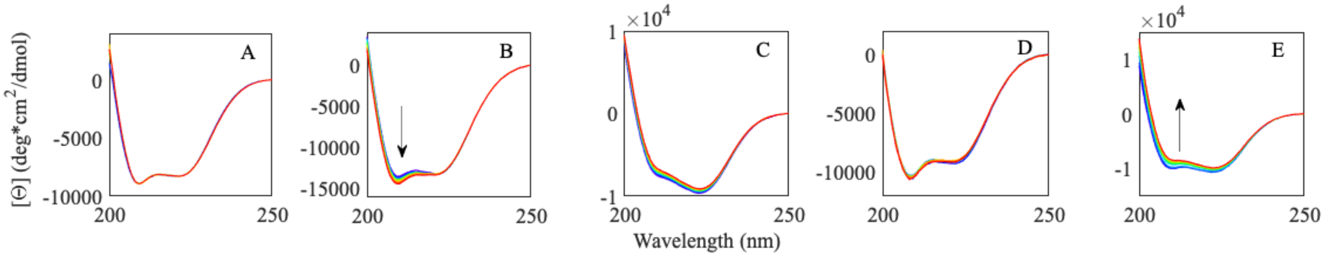


**Figure S6.** Averaged secondary structural CD spectra of free BSA (A), BSA exposed to 0 h PE (B), BSA exposed to 12 h PE (C), BSA exposed to 24 h PE (D) and BSA exposed to 48 h PE (E). All samples were prepared to 7 µM protein in either 10 mM Phosphate buffer or 100 mM HEPES (BSA), pH 7. Data was collected every 4 minutes over the course of 60 minutes (blue → green → red). Arrows denote CD spectral shift over the probe time.

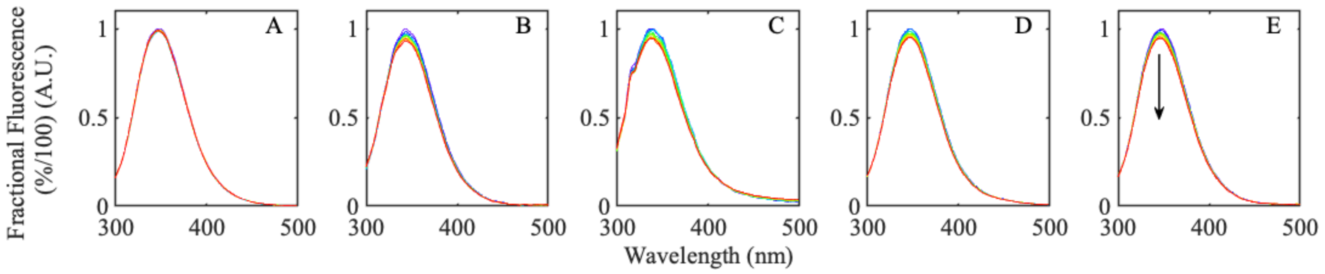


**Figure S7**. Averaged W-fluorescence spectra of free BSA (A), BSA exposed to 0h PE (B), BSA exposed to 12h PE (C), BSA exposed to 24h PE (D), BSA exposed to 48h PE (E) All samples were prepared to 7 µM protein in 10 mM Phosphate buffer, pH 7. Data was collected every 5 minutes over the course of 60 minutes (blue → green→ red). Arrows denote fluorescence spectral shift over the probe time.

### *Additional Fluorescence Analysis Discussion*

Fractional fluorescence intensity was calculated for all BSA and BSA-PE species and showed a decrease in percent intensity in all BSA exposed to PE (Figure 6A). All BSA-PE samples show evidence of binding kinetics and can be fit using a langmuir binding model. Clear 2-state transitions show up again in the 12 h PE samples. The 3-state system reappears in 0 h and 48 h samples, with local minima occurring around 21 minutes. Conversely, it is not clear whether 24 h reflects a standard 2- or 3- state system. The kinetics extracted from 2- or 3- states models is not as critical here as the necessity of more than one model to describe the system. Because of the difference in degradation landscape on the surface of the PE samples, it is not surprising that different models were required to describe degraded PE samples. The variation in data behavior strongly supports a requisite dynamic response of the protein to initiate polymer surface binding.

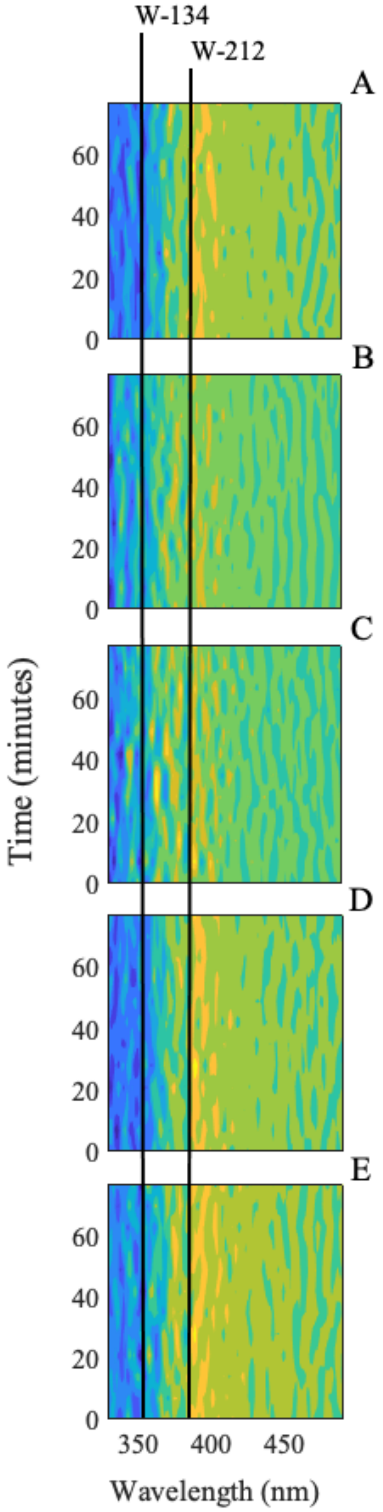


**Figure S8.** Second derivative analysis of averaged W-fluorescence of free BSA (A), BSA-0 h PE (B), BSA-12 h PE (C), BSA-24 h PE (D), BSA-48 h PE (E), over the course of one hour. Thermal mapping is reported from negative to zero value (blue →green→ yellow).

**Table S2.** Summary of observed changes in BSA structures interpreted from CD spectra

| Polymer | CD | Fractional helicity | Global motion |
|---|---|---|---|
| Free BSA | No change | No change | |
| 0 h | More helix | No change | Helical tightening |
| 12 h | Less helix | Less helix | Helical unfolding |
| 24 h | No change | Less helix | Local unfolding of helical end caps |
| 48 h | Less helix | Less helix | Helical Unfold |

**Table S3.** Summary of changes to the polymer characteristics referenced to pristine (0 h) PE

| Polymer Degradation Time | Surface Crystallinity | Bulk Crystallinity | Carbonyl Index | Superficial change |
|---|---|---|---|---|
| 0 h | -- | -- | -- | -- |
| 12 h | increase | increase | increase | Total polymer degradation |
| 24 h | Slight increase | decrease | increase | Structural rearrangement, incorporating O into surface? |
| 48 h | increase | increase | increase | Continued Degradation |

**Table S4.** Summary of observed changes in BSA structures interpreted from fluorescence spectra

| Polymer | SDA | Peak Fluorescence | COM | W environment |
|---|---|---|---|---|
| Free BSA | No change | No change | No change | |
| 0 h | W-212 decreases | decrease | decrease | W-212 hydrates, W134 becomes more buried |
| 12 h | W-212 decreases | decrease | Increase | w-212 hydration drives fluorescence shift |
| 24 h | W-212 recovers | Decrease | Decrease | W-212 hydrates, W134 becomes more buried |
| 48 h | W-212 hydrates | decrease | decrease | W-212 hydrates, W134 becomes more buried |

**Table S5.** Summary results from polymer characterization and interpretation of CD (global) and fluorescence (local) spectra

| Sample | Surface | Global | Local |
|---|---|---|---|
| Free BSA | -- | | |
| 0 h | -- | Helical tightening | W-212 hydrates, W134 becomes more buried |
| 12 h | Total polymer degradation | Helical unfolding | w-212 hydration drives fluorescence shift |
| 24 h | Structural rearrangement, incorporating O into surface? | Local unfolding of helical end caps | W-212 hydrates, W134 becomes more buried |
| 48 h | Continued Degradation | Helical Unfold | W-212 hydrates, W134 becomes more buried |

**REFERENCES:**

(1) Wei, Y.; Thyparambil, A. A.; Latour, R. A. Protein helical structure determination using CD spectroscopy for solutions with strong background absorbance from 190 to 230nm. *Biochimica et Biophysica Acta (BBA) - Proteins and Proteomics* **2014**, *1844* (12), 2331-2337. DOI: https://doi.org/10.1016/j.bbapap.2014.10.001.

(2) Tigner, J. M.; Elmer-Dixon, M. M.; Maurer-Jones, M. A. Quantification of Polymer Surface Degradation Using Fluorescence Spectroscopy. *Anal. Chem.* **2023**, *95* (26), 9975-9982. DOI: 10.1021/acs.analchem.3c01151.

(3) Majorek, K. A.; Porebski, P. J.; Dayal, A.; Zimmerman, M. D.; Jablonska, K.; Stewart, A. J.; Chruszcz, M.; Minor, W. Structural and immunologic characterization of bovine, horse, and rabbit serum albumins. *Mol. Immunol.* **2012**, *52* (3), 174-182. DOI: https://doi.org/10.1016/j.molimm.2012.05.011.
